# Salvianolic acid B attenuates cellular senescence and age-related decline in muscle function via dual mTOR/TP53INP2-autophagy regulation

**DOI:** 10.3389/fchem.2026.1771968

**Published:** 2026-03-05

**Authors:** Kaixin Liang, Mengying Hu, Hejing Zhao, Waner Xu, Yihan Zhang, Ruikun He, Cheng Peng, Hansen Chen, Zhenyu Ju, Shu Wu, Yuanlong Ge

**Affiliations:** 1 State Key Laboratory of Bioactive Molecules and Druggability Assessment, Guangdong Basic Research Center of Excellence for Natural Bioactive Molecules and Discovery of Innovative Drugs, College of Life Science and Technology, Jinan University, Guangzhou, Guangdong, China; 2 Key Laboratory of Regenerative Medicine of Ministry of Education, Institute of Aging and Regenerative Medicine, Department of Developmental & Regenerative Medicine, College of Life Science and Technology, Jinan University, Guangzhou, Guangdong, China; 3 BYHEALTH Institute of Nutrition & Health, Guangzhou, China; 4 Department of Geriatrics, National Key Clinical Specialty, Guangzhou First People’s Hospital, School of Medicine, South China University of Technology, Guangzhou, China

**Keywords:** Autophagy, cellular senescence, muscle, Salvianolic acid B, SASP

## Abstract

**Background:**

The rapid aging of the global population and the high prevalence of age-related diseases have positioned anti-aging as a critical research priority. Natural products have recently gained significant attention in anti-aging research owing to their multi-target and multi-pathway mechanisms, high safety profiles, and substantial potential for practical ap-plications.

**Methods:**

We performed *in vitro* experiments using aging fibroblasts (BJ) and identified the natural product Salvianolic acid B (SAB) as a potential anti-aging agent, based on its ability to reduce the expression of IL6 and IL1B. Furthermore, we assessed the effects of SAB on cell viability, proliferation, aging-related markers, SASP, ROS levels, and ATP pro-duction. To further investigate the underlying mechanisms of SAB’s anti-aging effects, we utilized network pharmacology and RNA sequencing analyses. *In vivo*, we used a mouse model of radiation-induced premature aging to evaluate the efficacy of SAB in alleviating age-related decline in muscle function, employing kinematic experiments and histological assessments.

**Results:**

Research has demonstrated that SAB effectively alleviates the cellular senescence phenotype. It was observed that SAB reduces SASP expression through modulation of the mTOR pathway. Additionally, SAB was found to decrease cellular ROS levels and enhance ATP production. *In vivo* studies further revealed that SAB ameliorates age-related decline in muscle function, potentially through promoting TP53INP2 expression to enhance autophagy. These findings provide novel insights into the potential applications of SAB in the field of anti-aging research.

**Conclusion:**

The study demonstrates that SAB exerts beneficial effects on cellular aging markers, including SASP, ROS, and ATP levels, and alleviates age-related decline in muscle function.

## Graphical Abstract

**Figure:**
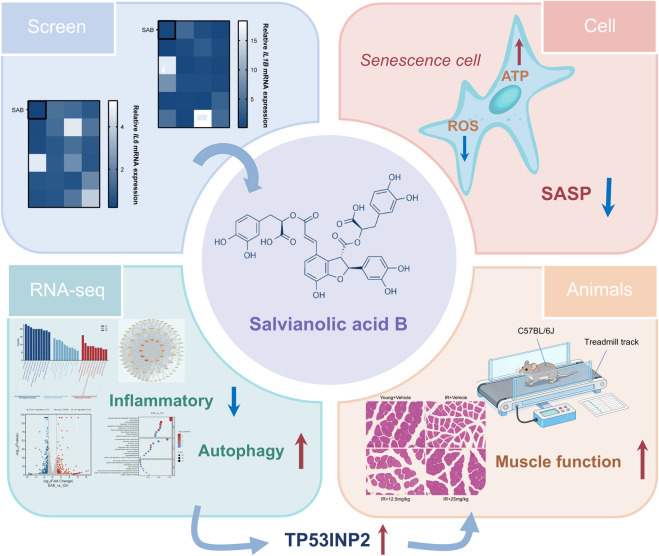

## 1 Introduction

The global population is undergoing a significant aging trend. with the number and proportion of elderly individuals increasing in many countries worldwide (WHO. Ageing and health, 2024). As aging progresses, the tissues and organ functions of elderly individuals deteriorate, leading to an increased susceptibility to age-related diseases such as diabetes, Alzheimer’s disease, and sarcopenia (Campisi et al., 2019). The increasing prevalence of age-related diseases shortens healthy lifespan, highlighting the importance of anti-aging research in modern science. Previous research indicates that cellular senescence impacts bodily tissues through two primary mechanisms (Childs et al., 2015). Firstly, the accumulation of senescent cells directly impacts the tissue or organism in a negative manner. Secondly, senescent cells secrete a large quantity and a variety of inflammatory factors known as the senescence-associated secretory phenotype (SASP) (Gasek et al., 2021). These inflammatory factors propagate the senescent phenotype to surrounding cells by altering the composition of the extracellular matrix, ultimately leading to systemic chronic inflammation and the development of age-related diseases, a phenomenon commonly referred to as inflamm-aging (Li et al., 2021; Li et al., 2024a). Therefore, two main strategies related to senescence are commonly employed: the clearance of senescent cells and the inhibition of SASP expression (Di Micco et al., 2021; Zhang et al., 2023). Recent studies have demonstrated that procyanidin C1 (PCC1) even exhibits anti-senescent effects by suppressing SASP formation at low concentrations, while selectively eliminating senescent cells at higher concentrations (Xu et al., 2021). Although the clearance of senescent cells has demonstrated beneficial effects in mitigating age-related diseases (Baker et al., 2011), it is important to recognize the limitations of this approach. While senescent cells are often implicated in age-related pathologies, they also contribute positively to processes such as embryogenesis, anti-tumor defense, and tissue repair (Calcinotto et al., 2019). Consequently, the clearance of senescent cells may remove both their harmful and beneficial effects, leading to ongoing controversy regarding this therapeutic strategy. In light of these considerations, current research is increasingly directed toward modulating the phenotype of senescent cells to counteract aging, including strategies such as inhibiting SASP expression, enhancing autophagy (Aman et al., 2021), and improving mitochondrial function (López-Otín et al., 2023).

Currently, numerous drugs exhibit anti-aging effects, among which natural products have emerged as a prominent research focus in anti-aging studies due to their unique advantages. Classic examples include resveratrol, curcumin, and quercetin (Yang et al., 2020) (Kirkland and Tchkonia, 2020; Matacchione et al., 2021). Recent discoveries include Mitophagy-inducing coumarin (MIC), which promotes mitophagy and extends the lifespan of *Caenorhabditis elegans* by inhibiting ligand-induced activation of the nuclear hormone receptor DAF-12/FXR (Chamoli et al., 2023). Oleuropein, an activator of the mitochondrial calcium uniporter (MCU), enhances mitochondrial calcium uptake by binding to the MICU1 subunit of MCU, thereby improving endurance and reducing fatigue in both young and aged mice (Gherardi et al., 2024). Among these natural products, many are derived from diverse sources, including plants, animals, and microorganisms (Ekiert and Szopa, 2020), and contain multiple active components that target various biological pathways, potentially resulting in comprehensive anti-aging effects. Moreover, compared to synthetic drugs, natural products typically exhibit lower toxicity and fewer side effects, making them safer and more suitable for long-term use. Thus, natural products represent a valuable and ongoing resource for drug development.

In this study, we aimed to identify novel natural products with potential anti-aging properties. Using IL6 and IL1B as markers of the SASP, we screened a range of natural compounds. Salvianolic acid B (SAB) emerged as a promising candidate, SAB is a major bioactive phenolic acid compound extracted from the dried root and rhizome of Salvia miltiorrhiza Bunge (Danshen), a classic medicinal and edible traditional Chinese medicine (TCM) with a long history of clinical application in cardiovascular and anti-inflammatory therapies (Li et al., 2020; Li et al., 2024b). Notably, SAB has completed a phase I clinical trial in healthy Chinese volunteers, demonstrating favorable safety, tolerability, and predictable pharmacokinetics—with no severe adverse events reported even at therapeutic doses (Cheng et al., 2023). Preclinical studies have further confirmed SAB’s multi-target pharmacological activities, including anti-oxidation (Sun et al., 2024), anti-inflammation (Wei et al., 2025), and autophagy regulation (Chen et al., 2023; Sun et al., 2022; Xiang et al., 2022; Zhao et al., 2019), which align with the core hallmarks of aging targeted in this research. As a natural product derived from a medicinal and edible TCM, SAB combines the advantages of high biocompatibility, low toxicity, and clinical translatability, acting as a senomorphic modulates senescent cell function without eliminating them—demonstrating the ability to reduce SASP expression, enhance mitochondrial function, and promote autophagy in senescent cells. RNA-seq analysis revealed that SAB treatment significantly upregulated TP53INP2, a gene associated with promoting autophagy and alleviating age-related sarcopenia (Sebastián et al., 2024). To validate these findings, we employed a premature aging mouse model and administered SAB via intraperitoneal injection, observing significant improvement in age-related decline in muscle function.

## 2 Materials and methods

### 2.1 Preparation of Salvianolic acid B

Salvianolic acid B was purchased from Selleck (#S4735, HPLC: 99.73%).

### 2.2 Senescent cell model

Construction of the replicative senescent cell model involved continuous passaging of BJ cells until 45 PD (Population Doubling), at which point the cells lost their proliferative capacity and were used for subsequent processing; low-passage BJ cells (20–25 PD) were used as the control group. For the construction of the irradiation-induced senescent cell model, low-passage BJ cells (20–25 PD) were exposed to X-rays at a dose of 10 Gy and 25 mA using a Rad Source Technologies (United States) X-ray irradiator. Subsequently, the irradiated cells were cultured for 1-week post-irradiation to allow senescence induction before further processing.

### 2.3 Cell culture and treatment

The BJ and HAEC cells were purchased from Chinese Academy of Sciences of Type Culture Collection (CTCC). In an incubator with 5% CO2, the cells were cultivated in DMEM supplemented with 10% fetal bovine serum, 1% penicillin/streptomycin, and 37 °C. Salvianolic acid B (Selleck, United States) were dissolved in DMSO to form a reserve solution. 100 μM and 200 μM Salvianolic acid B were utilized in BJ and HAEC cells for 24 h. A parallel DMSO vehicle control group was included, with the final DMSO concentration in all groups controlled at ≤ 0.2% (v/v) to exclude solvent-related interference.

### 2.4 Accelerated aging mouse model

8-week-old male C57BL/6J mice were purchased from GemPharmatech Co.,Ltd. Under SPF conditions, five mice per cage were allowed a 12-h light-dark cycle. All animal experiments were conducted following the guidelines and protocols approved by the Animal Protection and Ethics Committee of Jinan University (approval number 20240428-10). To establish a premature aging mouse model, X-rays (Rad Source Technologies, United States) were used at a dose of 4.5 Gy, 25 mA, followed by normal feeding for 2 months post-irradiation. After 2 months, SAB (Selleck, United States) was administered via intraperitoneal injection every 2 days for a total duration of 28 days.

### 2.5 Cell viability assays

Cell viability experiments were conducted using the CCK-8 assay kit (Beyotime, China). Cells were seeded at a density of 4,000 cells per well in a 96-well plate and incubated at 37 °C in a cell culture incubator for 24 h. After 24 h, the corresponding solvent or SAB (Selleck, United States) was added to each well and incubated at 37 °C in the cell culture incubator for another 24 h. Following this, the cell culture medium was aspirated from the 96-well plate, and 100 μL of 10% CCK-8 reagent was added to each well, followed by incubation of the cells at approximately 1 h at 37 °C in the cell culture incubator. Subsequently, the absorbance values were measured at a wavelength of 450 nm using a microplate reader (Biotek, United States) to assess cell viability. Cell viability (%) was calculated using the formula: (Experimental group absorbance value - Background absorbance value)/(Control group absorbance value - Background absorbance value) × 100%.

Briefly, cells were seeded at a density of 5 × 10^4^ cells per well and allowed to adhere for 24 h. They were then treated with the corresponding solvent or SAB under identical conditions to the CCK-8 assay. After 24 h of treatment, the medium was aspirated, and cells were washed twice with pre-warmed PBS. Subsequently, 0.5 mL of 0.25% trypsin-EDTA was added to each well and incubated at 37 °C for 3–5 min until complete detachment. Digestion was terminated by adding 1 mL of complete DMEM medium, and a single-cell suspension was prepared by gentle pipetting. A 10 μL aliquot of the cell suspension was analyzed using an automated cell counter to determine the viable cell concentration. Collect cells and wash with PBS, centrifuge and discard the supernatant. Incubate cells with DCFH-DA (Beyotime, China) in serum-free medium at 37 °C for 30 min. Subsequently, wash the cells three times with serum-free medium, and detect cellular ROS using flow cytometry.

To control for potential confounding effects of mitochondrial metabolic activity on the interpretation of CCK-8 results, a cell counting assay was performed. The experiment was under the same grouping and treatment conditions as the CCK-8 assay.

### 2.6 Measurement of intracellular ATP

The intracellular ATP levels were determined utilizing an ATP assay kit (Beyotime, China). BJ cells were lysed as per the manufacturer’s guidelines. Specifically, cells in a 6-well plate were lysed in 200 μL of lysis buffer, followed by centrifugation at 4 °C and 12,000×g for 5 min. Subsequently, 20 μL of the resulting supernatant was utilized for ATP quantification. The ATP concentration was assessed using a microplate reader from (Biotek, United States), normalized to nmol/mg of protein, and the relative ATP levels were calculated.

### 2.7 Western blot

Proteins were extracted using RIPA lysis buffer (Beyotime) containing 1% protease and phosphatase inhibitor cocktails (MCE). Protein concentrations were determined by BCA assay (Thermo Fisher), and 30 μg of each sample was separated by SDS-PAGE for proteins of interest. Electrophoresis was conducted at 80 V for 30 min followed by 120 V for 90 min. Proteins were transferred to PVDF membranes (Millipore) via wet transfer in Tris-glycine buffer with 20% methanol at 90 V (60 min) for low-molecular-weight proteins or 100 V (90 min) for larger proteins. After blocking with 5% BSA for 1 h, membranes were incubated with primary antibodies against LC3B (1:1,000, Proteintech), p62 (1:1,000, MCE), β-actin (1:200,000, Abclonal), p-mTOR(1:1,000,CST), p-S6K(1:1,000, CST), 4E-BP1 (1:1,000, CST) and GAPDH (1:200,000, Abclonal) at 4 °C overnight, followed by HRP-conjugated secondary antibody (1:2000, CST) incubation for 2 h at 37 °C. Signals were detected by ECL (Millipore) and quantified using ImageJ after imaging with a ChemiDoc XRS + system (Bio-Rad).

### 2.8 RNA extraction and real-time RT-PCR

Total RNA was extracted from cultured BJ cells (proliferative, replicative senescent, or irradiation-induced senescent) using Trizol reagent (Takara) immediately after harvesting from culture dishes (24 h post-SAB or vehicle treatment). Subsequently, 1 μg of RNA was utilized for cDNA synthesis utilizing a First-Strand cDNA Synthesis kit following the manufacturer’s protocols (Vazyme, China). Real-time RT-PCR analysis was conducted using the Real-Time System (Thermo Fisher, Waltham, United States) and SYBR Green Mix. The relative expression levels of the target genes were determined using the relative delta-delta-Ct method.

### 2.9 Network pharmacology analysis

Using the keyword “Salvianolic acid B,” relevant target points were searched in the Herb, GeneCards, and Swiss Target Prediction databases. Similarly, using the keyword “Senescence cell,” relevant target points were searched in the GeneCards database. The identified target points were then intersected to find common interacting genes. Subsequently, the common genes were inputted into the STRING database (version 12.0) to construct a protein-protein interaction (PPI) network with “*Homo sapiens*” as the reference species. The resulting network was visualized using Cytoscape 3.7.1 software. GO pathway enrichment analysis of the intersecting targets was performed using the clusterProfiler(4.6.2) R package.

### 2.10 RNA-seq analysis

The experiment was outsourced to Novogene Bioinformatics Technology Co. Ltd. Transcriptome sequencing data were analyzed for differential gene expression using the DESeq2 package (v 1.38.3). The volcano plot was utilized to display the numbers of upregulated and downregulated genes, while the heatmap was employed to illustrate the trends of gene upregulation and downregulation. Gene Ontology (GO) and Kyoto Encyclopedia of Genes and Genomes (KEGG) pathway enrichment analyses were conducted using the clusterProfiler package (v 4.6.2) in conjunction with the org. Hs.e.g.,.db (3.16.0) and org. Mm.e.g.,.db (3.16.0) R packages.

### 2.11 Treadmill endurance determination

The treadmill (Anhui Zhenghua Biological Instrument Equipment Co., Ltd., China) was positioned at a 15° angle relative to the ground. Prior to the formal treadmill testing, mice underwent continuous running adaptability training for 3 days, with the following running speed and duration: 5 rpm for 2 min, 7 rpm for 2 min, and 9 rpm for 1 min. During the actual experiment, the initial running speed for the mice was set at 5 rpm, with an increase of 2 rpm every 2 min until the mice reached exhaustion, and the distance run by the mice was recorded. Exhaustion was defined as the mice stopping running for 3 s or more even under electrical stimulation. The work done by the mice during running (kJ) was calculated as the product of the mouse’s body weight (kg), the distance run by the mouse (m), gravitational acceleration (g = 9.8 m/s^2), and sin(15°).

### 2.12 Grip strength determination

The mouse’s limbs were placed on a grip strength meter grid (Shanghai Xinruan, China), and the mouse’s tail was gently pulled backward until the mouse’s limbs detached from the grid. The grip strength meter recorded the instant grip force (gf) at the moment when the mouse’s limbs detached from the grid.

### 2.13 HE staining

Fresh muscle tissues were fixed in 4% paraformaldehyde for 24 h, followed by dehydration in a graded alcohol series. The tissues were embedded in paraffin blocks for sectioning. Muscle tissues were sliced into 4 µm thick sections. After dewaxing paraffin sections, the nuclei were stained with hematoxylin and the cytoplasm with eosin. Following staining, the sections were dehydrated, sealed, and dried to obtain image information. Subsequently, the sections were scanned using panoramic scanning (3D HISTECH, Hungary) to acquire high-definition images for observation and analysis.

### 2.14 SA-β-gal staining

Cells were fixed for 15 min at room temperature using 4% paraformaldehyde solution, followed by washing the cells with PBS. The cells were then treated with a staining solution containing X-gal according to the instructions provided by SA-β-Gal assay kit (Beyotime, China). After staining, the cells were incubated at 37 °C for 12 h. Following incubation, the cells were washed with PBS to remove unreacted substrates and stains. Microscope was used to observe the cells, and the positivity rate was calculated from 100 cells per group.

### 2.15 5-Ethynyl-2′-deoxyuridine (EdU) growth assay

The cells were exposed to a culture medium containing EdU for 4 h. After the incubation, the cells were gently washed with 3% BSA to remove unbound EdU. The cells were then permeabilized using PBS containing 0.3% Triton X-100. Subsequently, the cells were fixed for 15 min at room temperature using 4% paraformaldehyde solution. After washing, the cells were treated with the reaction mixture from an EdU staining kit (Beyotime, China) and incubated in the dark for 30 min. Following the washing of the cells, the cells were stained with Hoechst. T for 10 min. The cells were observed under a fluorescence microscope, and the positivity rate was calculated from 3 non-overlapping fields per group.

### 2.16 Statistical analysis

Data were analyzed using GraphPad Prism (Version 9.0.0, United States) and are shown as means ± standard deviations. An unpaired 2-tailed Student’s t-test was used to compare two experimental groups. Differences in three groups were assessed by a one-way ANOVA test. ns, not significant, *p < 0.05, **p < 0.01, ***p < 0.001, ****p < 0.0001.

## 3 Results

### 3.1 Screening of natural compounds with the potential to inhibit SASP

**FIGURE 1:**
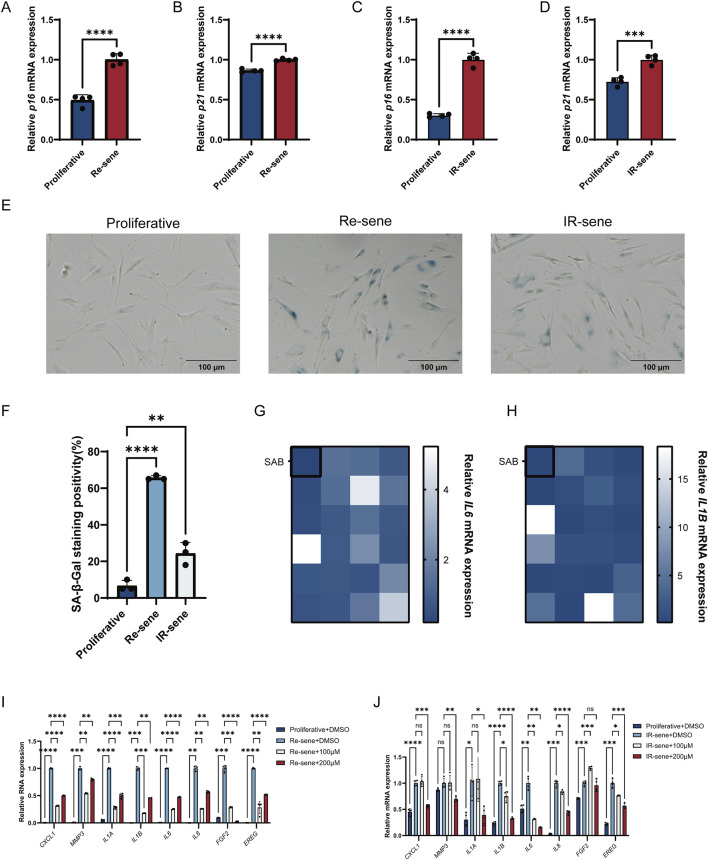
Screening of natural compounds with the potential to inhibit SASP. **(A,B)** The mRNA expression levels of p16 and p21 in replicative senescent cells; n = 4. **(C,D)** The mRNA expression levels of p16 and p21 in irradiation-induced senescent cells; n = 4. **(E)** Representative images displaying SA-β-Gal staining in Proliferative, Re-sene and IR-sene groups. Proliferative, Proliferative cells; Re-sene, replicative senescent cells; IR-sene, irradiation-induced senescent cells. Scale bars, 100 μm; n = 3 **(F)** The proportion of SA-β-gal-positive cells in Proliferative, Re-sene and IR-sene groups. **(G,H)** The mRNA expression levels of IL6 and IL1B in Replicative senescent cells after treatment with 24 different natural compounds for 24 h **(I)** mRNA expression levels of multiple SASP factors in Proliferative, Re-sene, Re-sene+100 μM, and Re-sene+200 μM groups in BJ cells (normal human skin fibroblasts). Re-sene+100 μM: replicative senescent BJ cells treated with 100 μM SAB for 24 h; Re-sene+200 μM: replicative senescent BJ cells treated with 200 μM SAB for 24 h; n = 4. **(J)** The mRNA expression levels of multiple SASP factors in Proliferative, IR-sene, IR-sene+100 μM and IR-sene+200 μM groups. IR-sene+100 μM, irradiation-induced senescent cells with treatment with 100 μM SAB for 24 h; IR-sene+200 μM groups, irradiation-induced senescent cells with treatment with 200 μM SAB for 24 h; n = 4.

To identify new compounds that can effectively modulate senescent cells, we screened 24 natural compounds. We utilized a normal human skin fibroblast cell line, BJ, and human aortic endothelial cells, HAEC, as a cellular model for this study. Cellular senescence was confirmed by the upregulation of p16 and p21 mRNA expression (Figures 1A–D) and increased SA-β-Gal staining (Figures 1E,F). During the screening phase, replicative senescent cells were treated with various natural compounds for 24 h. Based on the reduced expression of typical SASP factors, IL6 and IL1B, SAB was identified as a potential anti-aging compound (Figures 1G,H). To further validate the inhibitory effect of SAB on SASP in senescent cells, additional tests were performed on various SASP cytokines in replicative senescent cells (Figure 1I; Supplementary Figure S1A). Similar assessments were conducted on irradiation-induced senescent cells (Figure 1J). The results demonstrate that SAB suppresses SASP expression in both cell types.

### 3.2 The effects of SAB on various senescence phenotypes

**FIGURE 2:**
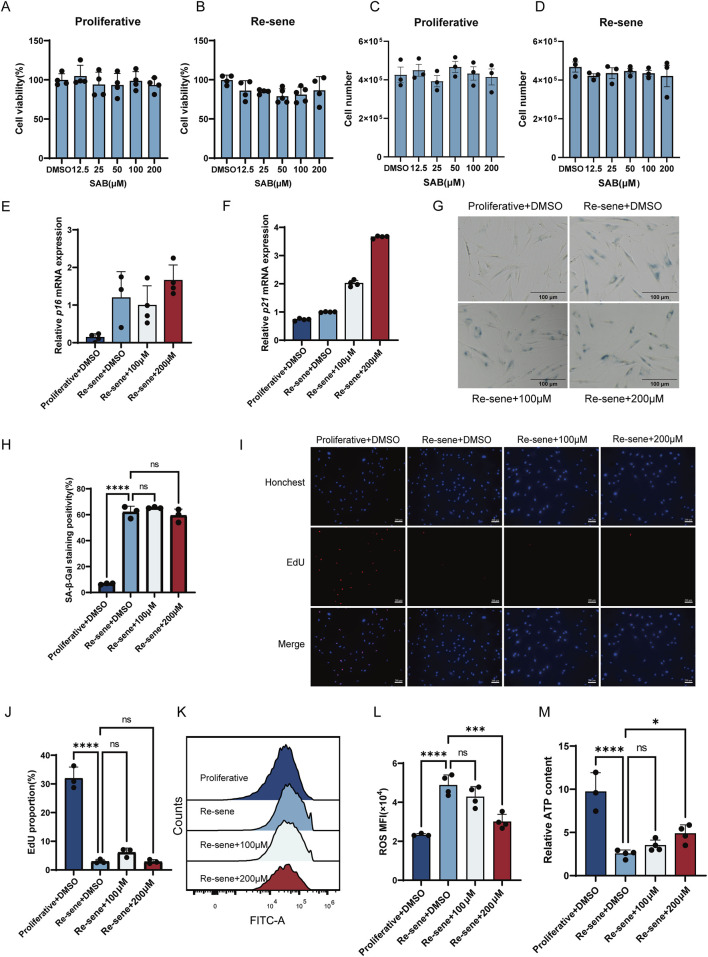
The effects of SAB on various senescence phenotypes. **(A–D)** Cell viability of proliferative and senescent BJ cells upon treatment with different concentrations of SAB; n = 3-4. **(E,F)** The mRNA expression levels of p16 and p21 after treatment of replicative and radiation-induced senescent cells with two different concentrations of SAB; n = 4. **(G)** Representative images displaying SA-β-Gal staining in Proliferative and replicative senescent cells treated with SAB; n = 3. **(H)** The proportion of SA-β-gal-positive cells in Proliferative and replicative senescent cells treated with SAB. **(I)** Representative image of EdU fluorescence staining after SAB treatment in replicative senescent cells. n = 3. **(J)** The proportion of EdU fluorescence staining after SAB treatment in replicative senescent cells. **(K)** Representative flow cytometry plots of intracellular ROS detection in Proliferative and replicative senescent cells treated with SAB; n = 4. **(L)** Quantitative analysis of intracellular ROS levels in Proliferative and replicative senescent cells treated with SAB. n = 4. **(M)** ATP content in Proliferative and replicative senescent cells treated with SAB. n = 4.

To explore the potential effects of SAB on other senescence phenotypes, we first performed CCK8 assays to determine the safe concentration range of SAB for both proliferative and senescent cells. The results of the CCK8 assays indicated that SAB at concentrations below 200 μM did not cause significant cytotoxicity in either proliferative or senescent cells (Figures 2A,B). This finding was corroborated by a cell counting assay performed to rule out confounding effects from altered metabolic activity (Figures 2C,D). For the subsequent cell experiments, SAB concentrations of 100 μM and 200 μM were selected. To evaluate cellular senescence markers, we examined the expression of p16 and p21, the activity of β-galactosidase, and the status of cell proliferation. It is showed that SAB treatment did not significantly reduce p16 and p21 levels (Figures 2E,F) or alter β-galactosidase activity (Figures 2G,H). Furthermore, no significant changes in cell proliferation rates were observed compared to untreated controls (Figures 2I,J). These findings indicate that SAB cannot rescue cell cycle arrest in senescent cells or reverse cellular senescence. Although SAB did not significantly rescue cell cycle arrest in senescent cells, it was observed to inhibit excessive ROS production, thereby mitigating ROS-induced damage (Figures 2K,L). Additionally, SAB enhanced cellular metabolism by increasing ATP production, providing the energy required for cellular functions (Figure 2M). The above results indicate that although SAB is unable to rescue cell cycle arrest or reverse cellular senescence, it does have a certain degree of effect in slowing the cellular senescence phenotype, which aids in alleviating chronic inflammation, improving mitochondrial function, and enhancing cellular metabolism. SAB ameliorates senescent phenotypes through dual regulation of mTOR-dependent SASP suppression and TP53INP2-mediated autophagy activation, with concurrent reduction of intracellular ROS and enhancement of mitochondrial ATP production. These effects are not limited to antioxidant activity but reflect coordinated targeting of core senescence pathways—consistent with *in vivo* improvements in muscle function and reduction of age-related inflammation. Unlike pure antioxidants that only scavenge ROS without addressing upstream senescence drivers, SAB directly modulates mTOR and autophagy, validating its role as a senomorphic compound.

### 3.3 SAB reduces the expression of SASP in senescent cells by inhibiting the mTOR protein

**FIGURE 3:**
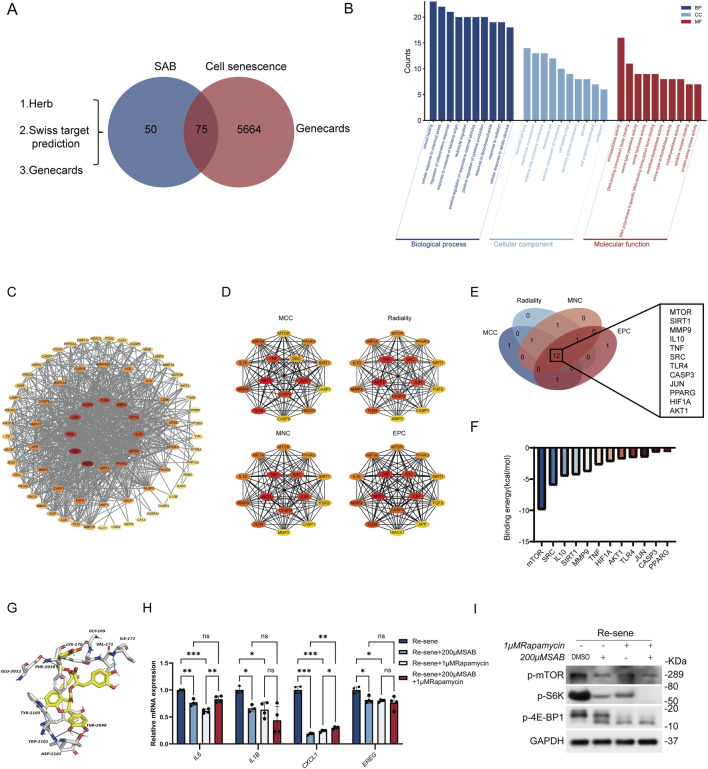
SAB reduces the expression of SASP in senescent cells by inhibiting the mTOR protein **(A)** Venn diagram of SAB and senescence-associated targets. **(B)** GO enrichment analysis. Counts (y-axis), term (x-axis); dark blue, light blue and red represent the 10 core results for BP, CC, and MF, respectively. **(C)** SAB anti-senescence PPI network. **(D)** Four computational approaches were individually employed to analyze the 15 targets most closely associated with SAB and cellular senescence. **(E)** Venn diagram of common targets derived from four computational methods. **(F)** The binding energy between SAB and various target molecules. **(G)** The simulated visualization model of the interaction between SAB and mTOR. **(H)** The mRNA expression levels of multiple SASP factors in Re-sene, Re-sene+200 μM SAB, Re-sene+1 μM Rapamycin and Re-sene+200 μM SAB +1 μM Rapamycin groups; n = 4. **(I)** Western blot analysis of phosphorylated mTOR, S6K, and 4E-BP1 in senescent BJ cells treated with SAB (100 μM, 200 μM) or rapamycin (1 μM). n = 3.

To identify the targets through which SAB downregulates SASP-related genes, we conducted systematic network analyses. We first retrieved SAB-related and senescence-associated targets from Herb, SwissTargetPrediction, and GeneCards databases, resulting in 50 SAB-related targets, 5,664 senescence-related targets, and 75 overlapping targets (Figure 3A). Subsequently, we conducted a GO analysis using these 75 targets, which indicated that SAB may influence the regulation of inflammatory responses (Figure 3B). To further elucidate the specific target on which SAB acts, we utilized STRING to analyze the associations between these 75 overlapping targets (Figure 3C). Red nodes indicate a more significant correlation between SAB and cellular senescence, whereas nodes in lighter shades represent a lesser or weaker correlation. To identify the most likely target of SAB action, we employed four distinct calculation methods to screen the top 15 targets (Figure 3D). Additionally, we selected the common genes among these four groups for molecular docking (Figure 3E). Binding energy analysis identified mTOR as the highest-affinity target for SAB (Figure 3F). Molecular modeling suggested SAB binding to mTOR at specific residues including GLY-169, LYS-170, VAL-171, ILE-172, GLU-2032, PHE-2039, THR-2098, TRP-2101, ASP-2102, and TYR-2105 (Figure 3G). To verify whether the inhibitory effect of SAB on SASP is dependent on mTOR, we used the mTOR inhibitor Rapamycin (Figure 3H). SASP suppression was achieved with either SAB alone or rapamycin alone. Combined treatment showed no additive inhibitory effect compared to individual agents. This non-additive effect suggests SAB-mediated SASP reduction operates through mTOR-dependent pathways. To further provide direct biochemical evidence for mTOR pathway inhibition, we performed Western blot analysis of key downstream effectors. The results demonstrated that SAB treatment (100 μM, 200 μM) dose-dependently reduced the phosphorylation levels of mTOR, S6K, and 4E-BP1 in senescent BJ cells, with an efficacy comparable to the rapamycin positive control (Figure 3I) Considering the high score of their combination, it is plausible that SAB can directly interact with mTOR to inhibit the mTOR’s effect in reducing the expression of SASP.

### 3.4 SAB effectively enhances the expression of TP53INP2 and promotes the occurrence of autophagy

**FIGURE 4:**
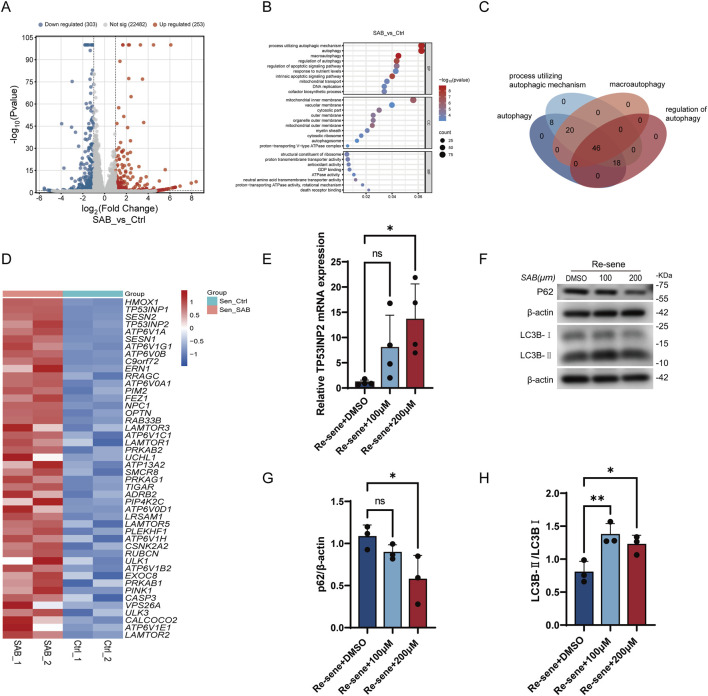
SAB effectively enhances the expression of TP53INP2 and promotes the occurrence of autophagy **(A)** Volcano plot of gene expression changes. **(B)** GO functional enrichment analysis. **(C)** Venn diagram of differentially expressed genes in autophagy-related pathways. **(D)** Heatmap of differentially expressed genes. **(E)** Expression level of TP53INP2; n = 4. **(F–H)** Expression levels of autophagy-related proteins. n = 3.

To investigate the mechanisms by which SAB ameliorates the senescence phenotype, we conducted RNA-seq analysis on senescent BJ cells treated with SAB. The results revealed that SAB treatment significantly upregulated 253 genes, downregulated 303 genes, and left 22,482 genes unchanged (Figure 4A). GO enrichment analysis of the significantly upregulated genes (Figure 4B) indicated that most changes were associated with autophagy-related pathways, including autophagy, processes utilizing autophagic mechanisms, macroautophagy, and regulation of autophagy. By intersecting the genes significantly altered in these four pathways, we identified 46 genes (Figure 4C). Among these 46 genes, TP53INP2, a gene involved in autophagy regulation, was identified (Figure 4D). Recent studies suggest that TP53INP2 is linked to muscle aging, and its upregulation in the muscles of aged mice promotes muscle cell autophagy, improving muscle function and exercise capacity. Therefore, we investigated whether SAB could enhance TP53INP2 expression and promote autophagy at the cellular level. Real-time quantitative PCR analysis confirmed that SAB treatment significantly increased TP53INP2 expression (Figure 4E). To further confirm the role of SAB in promoting cellular autophagy, we measured the protein expression levels of autophagy-related genes LC3B and p62. Western blot analysis revealed that SAB treatment significantly increased the LC3B-II/LC3B-I ratio and decreased p62 levels (Figures 4F–H). These results collectively indicate that SAB can effectively activate the cellular autophagy process. SAB targets senescent cells by enhancing TP53INP2-dependent autophagy (a key deficit in senescence), which in turn: (1) clears dysfunctional mitochondria to reduce ROS production and restore ATP metabolism; (2) suppresses mTOR-dependent SASP/inflammation; and (3) ultimately ameliorates age-related muscle dysfunction—without altering core senescence arrest markers (p16/p21, SA-β-Gal), consistent with its senomorphic identity. SAB ameliorates the muscle function and exercise capacity of aging mice.

**FIGURE 5:**
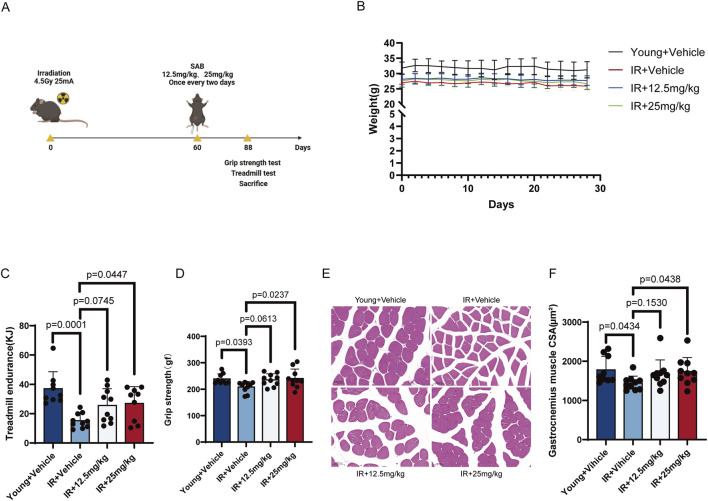
SAB ameliorates the muscle function and exercise capacity of aging mice **(A)** Diagram of animal experimental model. **(B)** Body weight curves; n ≥ 9. **(C)** Treadmill endurance test; n ≥ 9. **(D)** Mouse grip strength test; n ≥ 9. **(E)** Representative images of an H&E-stained gastrocnemius muscle; n ≥ 9. **(F)** Statistical chart of mouse gastrocnemius muscle cross-sectional area (CSA).

To evaluate the efficacy of SAB in improving muscle function and exercise capacity in aging mice, we first established a premature aging mouse model through irradiation (Figure 5A) and administered SAB via intraperitoneal injection every 2 day. After irradiation, the average body weight of premature aging mice was approximately 5 g lower than that of young mice. Following 1 month of drug intervention, no significant weight gain was observed in the premature aging mice (Figure 5B). Subsequently, we evaluated the exercise capacity of the mice. In the treadmill test (Figure 5C), we compared the exercise performance and energy expenditure between the premature aging mice and young mice. The results revealed a significant decline in exercise capacity and energy expenditure in premature aging mice. Compared to solvent-injected mice, low-dose SAB treatment showed a trend toward improved exercise capacity and increased energy expenditure, while high-dose SAB treatment significantly enhanced exercise capacity and energy expenditure. In the grip strength test (Figure 5D), similar results were observed: the grip strength of the premature aging mice was lower than that of the young mice, and after SAB injection, the grip strength of the low-dose group increased slightly, while that of the high-dose group was significantly greater than the solvent group. Furthermore, analysis of HE-stained sections revealed that the gastrocnemius muscle cross-sectional area (CSA) was significantly smaller in premature aging mice than in young mice (Figures 5E,F). After SAB treatment, the low-dose group showed an upward trend in muscle CSA, while the high-dose group exhibited a significantly larger CSA than the solvent group. These results collectively indicate that SAB can effectively enhance muscle function and exercise capacity in aging mice.

### 3.5 SAB mitigates premature aging-related muscle dysfunction and enhances exercise capacity in irradiation-induced premature aging mice

**FIGURE 6:**
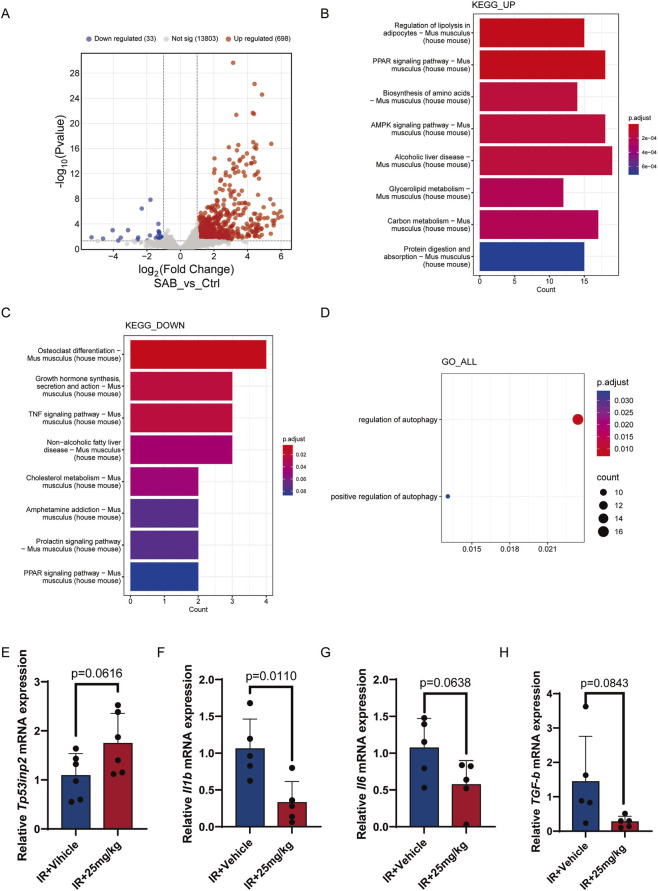
SAB decreases Inflammatory Factors in muscles and activates muscle autophagy **(A)** Volcano plot of gene expression changes. **(B)** KEGG enrichment analysis graph of significantly upregulated genes. **(C)** KEGG enrichment analysis graph of significantly downregulated genes. **(D)** GO enrichment analysis graph of significantly genes. **(E–H)** Relative mRNA expression of Tp53inp2, Il1b, Il6 and Tgfb; n ≥ 5.

To evaluate the efficacy of SAB in improving muscle function and exercise capacity in aging mice, we first established a premature aging mouse model through irradiation (Figure 5A) and administered SAB via intraperitoneal injection every 2 day. After intraperitoneal injection of SAB in mice, we conducted transcriptome sequencing analysis on the gastrocnemius muscle. The results revealed that 731 genes were significantly altered in the treated muscle tissue, including 33 downregulated and 698 upregulated genes (Figure 6A). KEGG enrichment analysis of the significantly upregulated genes identified the top 8 most significant pathways (Figure 6B), including the AMPK signaling pathway. Consistent with previous studies, activated AMPK is known to promote autophagy through multiple mechanisms. Additionally, AMPK enhances autophagy by inhibiting mTOR activity. Moreover, GO enrichment analysis of all significantly altered genes highlighted two pathways with significant changes: regulation of autophagy and positive regulation of autophagy (Figure 6D). Based on these findings, we propose that SAB can stimulate muscle autophagy. We also conducted KEGG enrichment analysis on the significantly downregulated genes and identified the top 8 most significant pathways (Figure 6C), including the TNF signaling pathway, suggesting that SAB may inhibit muscle inflammation. Furthermore, we measured the expression of Tp53inp2, a key autophagy regulator involved in muscle aging, and found that SAB treatment increased Tp53inp2 expression in mouse muscle (Figure 6E). We also analyzed SASP-related gene expression in muscle and observed that SAB treatment reduced the levels of Il6, Il1b, and Tgfb in mouse muscle (Figures 6F–H). These findings suggest that SAB ameliorates muscle aging by promoting autophagy through Tp53inp2 upregulation. Additionally, the anti-inflammatory and SASP-suppressing effects of SAB may contribute to the amelioration of muscle aging.

### 3.6 SAB decreases inflammatory factors in muscles and activates muscle autophagy

After intraperitoneal injection of SAB in mice, we conducted transcriptome sequencing analysis on the gastrocnemius muscle. The results revealed that 731 genes were significantly altered in the treated muscle tissue, including 33 downregulated and 698 upregulated genes (Figure 6A). KEGG enrichment analysis of the significantly upregulated genes identified the top 8 most significant pathways (Figure 6B), including the AMPK signaling pathway. Consistent with previous studies, activated AMPK is known to promote autophagy through multiple mechanisms. Additionally, AMPK enhances autophagy by inhibiting mTOR activity. Moreover, GO enrichment analysis of all significantly altered genes highlighted two pathways with significant changes: regulation of autophagy and positive regulation of autophagy (Figure 6D). Based on these findings, we propose that SAB can stimulate muscle autophagy. We also conducted KEGG enrichment analysis on the significantly downregulated genes and identified the top 8 most significant pathways (Figure 6C), including the TNF signaling pathway, suggesting that SAB may inhibit muscle inflammation. Furthermore, we measured the expression of Tp53inp2, a key autophagy regulator involved in muscle aging, and found that SAB treatment increased Tp53inp2 expression in mouse muscle (Figure 6E). We also analyzed SASP-related gene expression in muscle and observed that SAB treatment reduced the levels of Il6, Il1b, and Tgfb in mouse muscle (Figures 6F–H). These findings suggest that SAB ameliorates muscle aging by promoting autophagy through Tp53inp2 upregulation. Additionally, the anti-inflammatory and SASP-suppressing effects of SAB may contribute to the amelioration of muscle aging.

## 4 Discussion

This study elucidates the multifaceted roles and the underlying mechanisms of SAB in the regulation of cellular senescence. Our results demonstrate that SAB effectively inhibits SASP, potentially through suppression of the mTOR signaling pathway. Previous studies have reported that mTOR inhibition suppresses the SASP by specifically downregulating MAPKAPK2 translation (Herranz et al., 2015), while others have shown that mTOR inhibitors inhibit the SASP through suppression of IL-1α translation (Laberge et al., 2015) Based on these reported mTOR-regulated SASP mechanisms and our experimental results demonstrating that combined SAB-rapamycin treatment did not exhibit enhanced inhibitory effects compared to SAB alone, we hypothesize that SAB modulates SASP via inhibition of the mTOR pathway. Furthermore, inhibition of mTOR is known to activate autophagy, as demonstrated by the classical mTOR signaling pathway (Kim and Guan, 2015). This observation aligns with our aforementioned conclusion that SAB induces autophagy activation.

In cellular metabolism, SAB exhibits a dual effect by reducing ROS levels and enhancing ATP production. Previous research has shown that SAB can facilitate the translocation of NRF2 into the cell nucleus, thereby increasing the expression of antioxidant enzymes (Sun et al., 2024). This suggests that the ROS scavenging activity of SAB may stem from its regulation of NRF2, highlighting its potential to ameliorate mitochondrial dysfunction in senescent cells. Importantly, this ROS-modulating effect is not an isolated antioxidant action but part of SAB’s coordinated regulation of core senescence pathways—including mTOR-dependent SASP suppression and TP53INP2-mediated autophagy activation. Unlike pure antioxidants that only target ROS without addressing upstream senescence drivers, SAB’s multi-targeted action validates its role as a senomorphic compound and explains its ability to ameliorate comprehensive senescent phenotypes. The increase in ATP levels indicates that SAB may maintain cellular energy homeostasis by optimizing energy metabolism, thereby exerting anti-aging effects (Chu et al., 2021).

Although previous reports have indicated that SAB may exert its anti-aging effects by inhibiting the binding of SP1 to the promoters of P21/P16 and has been observed to reduce classical aging markers such as p16 and p21 in macrophages, cancer cells, and murine pulmonary fibrosis (Li et al., 2024b), our study did not detect any influence of SAB on the expression of these markers, nor did it alter β-galactosidase activity or EdU incorporation rates. This observation suggests that SAB may exert diverse effects through different mechanisms in various cells and tissues. Notably, the inability of SAB to reverse cell cycle arrest and proliferative cessation does not inherently contradict its anti-senescence potential. Current evidence indicates that cell cycle and proliferation arrest in senescent cells remains largely irreversible. Although a 2025 study by Youkun Bi et al. proposed a “Senoreverse” strategy to rescue proliferation arrest and rejuvenate senescent cells (SnCs) (Bi et al., 2025), the mechanistic basis for reversing such arrest remains poorly understood, with most current anti-senescence interventions failing to achieve this endpoint. Crucially, our findings demonstrate that SAB significantly ameliorates core senescent phenotypes (SASP suppression, ATP restoration, ROS reduction), thereby supporting its anti-senescence efficacy independent of cell cycle reprogramming.

In studies on the therapeutic applications of SAB, it is primarily used to treat various organ fibrosis (Fu et al., 2024; Li et al., 2024b) and cardiovascular diseases (Li et al., 2020; Wei et al., 2025), with no reported role in sarcopenia treatment. In our research for the mechanism of SAB in treating age-related sarcopenia, we found through RNA-seq that SAB promotes autophagy in both cells and muscles. Previous studies have shown that muscle ablation of the core autophagy protein ATG7 reduces muscle mass and causes neuromuscular junction degeneration in adult mice, indicating that autophagy in muscles decreases with age, and intervention can prevent muscle loss (Carnio et al., 2014) Therefore, we hypothesize that SAB enhances muscle function in aging by regulating the expression of the autophagy-related gene TP53INP2, thereby promoting muscle autophagy. Currently, sarcopenia treatment primarily relies on physical therapy, nutritional supplementation, and limited pharmacological interventions (Cho et al., 2022). Thus, the discovery of SAB’s effects offers a novel approach to sarcopenia treatment. Moreover, compared to existing treatment methods, SAB has some unique advantages. First, as a natural product, SAB exhibits high safety and minimal side effects, making it a gentler and safer alternative to some medications. Second, SAB treatment is simpler and more convenient than long-term physical therapy and nutritional supplementation, making it easier for patients to accept and adhere to the treatment plan. Notably, SAB has successfully completed a phase I clinical trial, demonstrating a favorable safety profile in human subjects (Cheng et al., 2023). This foundational evidence underscores its substantial translational potential as a therapeutic agent or nutraceutical formulation for sarcopenia prevention or intervention.

Our study has several limitations that need to be addressed. First, the primary mechanism by which SAB promotes autophagy, particularly regarding its direct influence on TP53INP2 expression versus a cofactor-dependent process, requires elucidation. Second, we used local injection as the administration method, and further evaluation of alternative routes, particularly oral administration, is needed to enhance treatment convenience for patients. Third, more research on the clinical safety, particularly hepatotoxicity as a common challenge in drug development (Chang and Schiano, 2007), and practicality of SAB is required to develop it into an effective health supplement or drug.

In summary, SABs exhibit significant anti-aging effects at the cellular level through their multi-targeted and multi-pathway mechanism of action. It also effectively ameliorates age-related decline in muscle function in animal models, making it a potential clinical intervention strategy. However, the specific targets, molecular mechanisms, and clinical research of SAB still require further in-depth study and refinement to provide a theoretical basis for developing more precise anti-aging treatments.

## Data Availability

The data that support the findings of this study are openly available in NCBI database at https://www.ncbi.nlm.nih.gov/sra/PRJNA1241894.
