# Supplementary material for: Salvianolic acid B attenuates cellular senescence and age-related decline in muscle function via dual mTOR/TP53INP2-autophagy regulation
Source: Front Chem. 2026 Mar 5;14:1771968. doi: 10.3389/fchem.2026.1771968 (PMC13000926; doi:10.3389/fchem.2026.1771968)
Supplement: Supplementary file 1 [file DataSheet1.pdf]

Supplementary Material

1 Supplementary Figures and Tables

For more information on Supplementary Material and for details on the different file types accepted, please see here.

1.1 Supplementary Figures

A

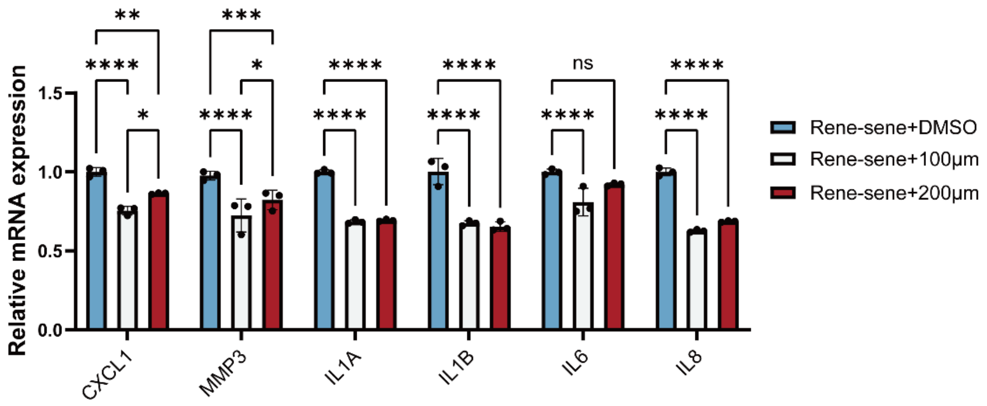

Supplementary Figure 1. mRNA expression levels of SASP factors in HAECs under SAB

(A) mRNA expression levels of multiple SASP factors in Proliferative, Re-sene, Re-sene+100 µM, a nd Re-sene+200 µM groups in human aortic endothelial cells (HAECs). Re-sene+100 µM: replic ative senescent HAECs treated with 100 µM SAB for 24 hours; Re-sene+200 µM: replicative sen escent HAECs treated with 200 µM SAB for 24 hours; n=3.

1.2 Supplementary Table

Table 1. Sequences of primers used for quantitative real-time PCR (qPCR)

Genus	Gene	Forward	Reverse
Human	*ACTB*	TTGCCGACAGGATG CAGAA	GCCGATCCACACGG AGTACT

	p16	AGCAGCATGGAGC CTTCGG	GCCCATCATCATGAC CTGGATCG
	p21	AGGTGGACCTGGAG ACTCTCAG	TCCTCTTGGAGAAG ATCAGCCG
	MMP3	CAAAACATATTTCTT TGTAGAGGACAA	TTCAGCTATTTGCTT GGGAAA
	CXCL1	AGCTTGCCTCAATCC TGCATCC	TCCTTCAGGAACAG CCACCAGT
	IL6	ACTCACCTCTTCAGA ACGAATTG	CCATCTTTGGAAGGT TCAGGTTG
	IL1B	TGCACGCTCCGGGA CTCACA	CATGGAGAACACCAC TTGTTGCTCC
2	*CXCL8*	ACTGAGAGTGATTG AGAGTGGAC	AACCCTCTGCACCCAG TTTTC
	IL1A	AGATGCCTGAGATAC CCAAAACC	CCAAGCACACCCAGTAGT CT
	fgf2	AGAGAGAGGAGTTG TGTC	GGTCCTGTTTGGATCCA
	EREG	TATCACAGTCGTCGG TTCCA	AACTCTGGATCCCCTGAG GTA

	TP53INP2	CGCCTCTCCAGCCTCTTCTTC	AGCCAGCCGTCCACTTCATC
Mouse	*18s*	CGCGGTTCTATTTTGTTGGT	AGTCGGCATCGTTTATGGTC
	Tp53inp2	CGCCTCCTGAAGACTCCAACTG	TATAGCTGTCCTGTAGGTCGATGATG
	Il6	CTTCTTGGGACTGATGCTGGTGAC	AGGTCTGTTGGGAGTGGTATCCTC
	Il1b	TCGCAGCAGCACATCAACAAGAG	AGGTCCACGGGAAAGACACAGG
	Tgfb	ACCGCAACAACGCCATCTATGAG	GGCACTGCTTCCCGAATGTCTG
